# Molecular and functional dissection of LIGULELESS1 (LG1) in plants

**DOI:** 10.3389/fpls.2023.1190004

**Published:** 2023-06-12

**Authors:** Lei Qin, Xintong Wu, Hang Zhao

**Affiliations:** ^1^ College of Life Sciences, Qufu Normal University, Qufu, China; ^2^ State Key Laboratory of Crop Biology, College of Agronomic Sciences, Shandong Agricultural University, Taian, China

**Keywords:** plant architecture, LG1, leaf angle, flower development, SBP

## Abstract

Plant architecture is a culmination of the features necessary for capturing light energy and adapting to the environment. An ideal architecture can promote an increase in planting density, light penetration to the lower canopy, airflow as well as heat distribution to achieve an increase in crop yield. A number of plant architecture-related genes have been identified by map cloning, quantitative trait locus (QTL) and genome-wide association study (GWAS) analysis. LIGULELESS1 (LG1) belongs to the squamosa promoter-binding protein (SBP) family of transcription factors (TFs) that are key regulators for plant growth and development, especially leaf angle (LA) and flower development. The DRL1/2-LG1-RAVL pathway is involved in brassinosteroid (BR) signaling to regulate the LA in maize, which has facilitated the regulation of plant architecture. Therefore, exploring the gene regulatory functions of *LG1*, especially its relationship with LA genes, can help achieve the precise regulation of plant phenotypes adapted to varied environments, thereby increasing the yield. This review comprehensively summarizes the advances in *LG1* research, including its effect on LA and flower development. Finally, we discuss the current challenges and future research goals associate with *LG1*.

## 1 Introduction

Plant growth and development are inextricably linked to the absorption of light energy (Ort et al., 2015). A reasonable leaf angle (LA) throughout different parts of the plant is crucial for making optimum use of solar energy and increasing biomass (Mantilla-Perez and Salas Fernandez, 2017). Specifically, plant varieties with narrow angles of the upper leaves promote an increased in planting densities and yields that is beneficial to meet the rising food demand (Cao et al., 2022b). For example, maize yields have increased more than 7-fold since the mid-twentieth century, primarily because of increased tolerance to high planting density and effectiveness of hybrids from increased heterosis (Duvick, 2005; Mansfield and Mumm, 2014; Wang et al., 2020).

**Figure 1:**
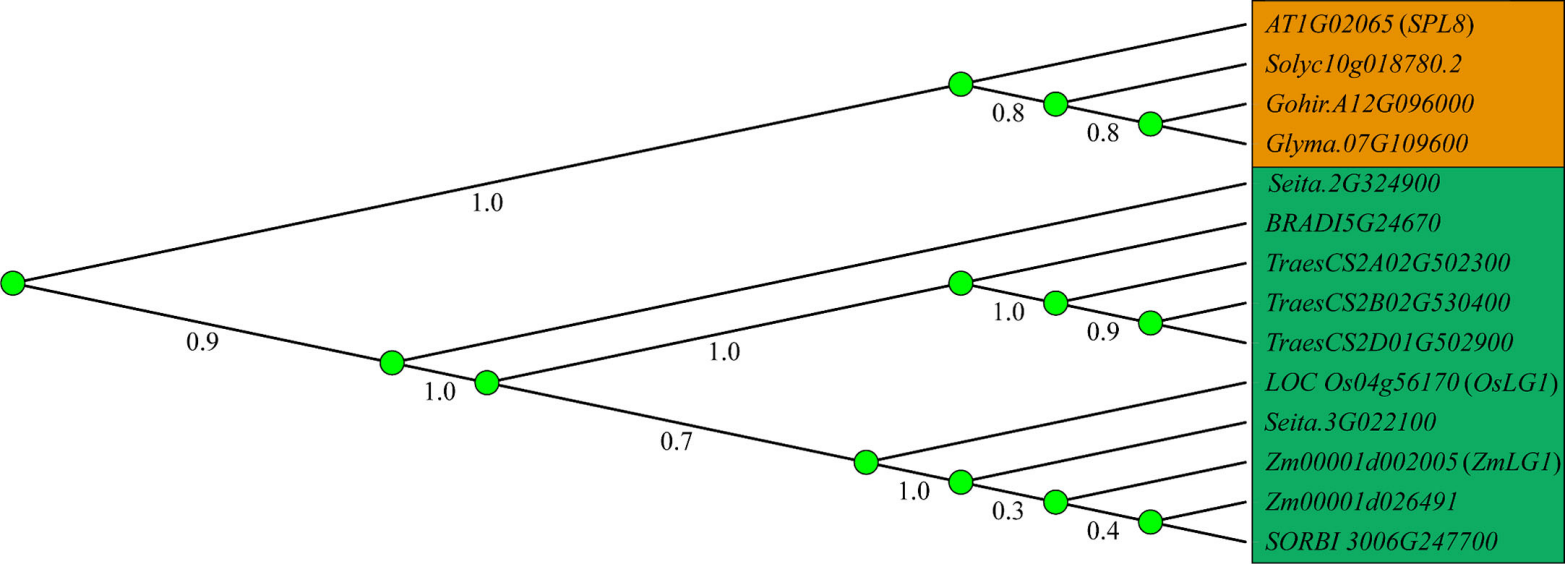
Phylogenetic relationship of *LG1* orthologs. Phylogenetic relationship of *LG1* in dicotyledon (orange) such as *Arabidopsis*, tomato, cotton, soybean, and monocotyledon (green) such as rice, maize, wheat, sorghum, *foxtail millet*, *Brachypodium*. The phylogenetic tree was created using MEGAX with LG1 orthologs sequences by neighbor-joining method, and 1000 bootstrap replicates.

Current advances in biotechnology have facilitated the study of the developmental and regulatory mechanisms of LA in plants. During the past three decades, *LG1* has been identified as the most critical transcription factor (TF) regulating LA and flower development (Moreno et al., 1997; Lewis et al., 2014). *LG1* is a member of the SBP family, which are special transcription factors in plants that contribute to growth and development as well as various physiological and biochemical processes (Klein et al., 1996; Cardon et al., 1999; Birkenbihl et al., 2005; Xie et al., 2006; Guo et al., 2008; Chen et al., 2010; Preston and Hileman, 2013; Zhang et al., 2016; Peng et al., 2019). SBP family proteins comprise 80 conserved amino acids, two conserved zinc finger domains (Zn1 and Zn2) and a C-terminal nuclear localization signal. The zinc finger domains specifically bind the GTAC core sequence in the downstream gene promoter, and SBP function is also conservatively regulated by miR156 throughout the growth period (Cardon et al., 1999; Birkenbihl et al., 2005; Guo et al., 2008; Chen et al., 2010; Peng et al., 2016). In *Arabidopsis*, overexpression of *AtSPL8*, a *ZmLG1* homologous gene, causes non-dehiscence of anthers through the gibberellin (GA) pathway and affects fertility (Zhang et al., 2007; Xing et al., 2010; Preston and Hileman, 2013) (**Figure 1**). In *maize*, *ZmLG1* affects the development of the ligular region. The homozygous *Zmlg1* mutant shows a phenotype with no ligule or auricles, thereby reducing the LA. It was later observed that *ZmLG1* is regulated by, for example, *ZmWAB1*and *ZmDRL1/2*, which affected the development of flowers and LA (Becraft et al., 1990; Sylvester et al., 1990; Becraft and Freeling, 1991; Moreno et al., 1997; Hay and Hake, 2004; Lewis et al., 2014; Tian et al., 2019). Recent studies have shown that *ZmLG1* is associated with resistance to northern leaf blight, which has improved the understanding of *ZmLG1* function (Kolkman et al., 2020). *ZmLG1* is involved in leaf morphological development, flower development and disease resistance, and its transcriptional and post-transcriptional regulation has received considerable attention. Hormone activity is critical for the development of the ligular region. Studies have found that *Zmlg1* mutants exhibit aberrant *ZmPIN1a* expression, and cannot form a continuous distribution in the preligule band (PLB) zone. However, there is no direct evidence that *ZmLG1* is involved in auxin signaling in maize (Johnston et al., 2014). In addition to auxin, ZmLG1 directly regulates *ZmRVAL1* to participate in the signaling involving brassinosteroids (BRs), which are the most important hormones shown to regulate LA (Tian et al., 2019). Based on the important role of *LG1* and the extensive research in recent years, we summarize the mechanisms of transcriptional and molecular regulation of LG1. We also valuate the challenges and the future prospects of *LG1* research.

## 2 Tissue-specific expression of *LG1* leads to various biological functions

**Figure 2:**
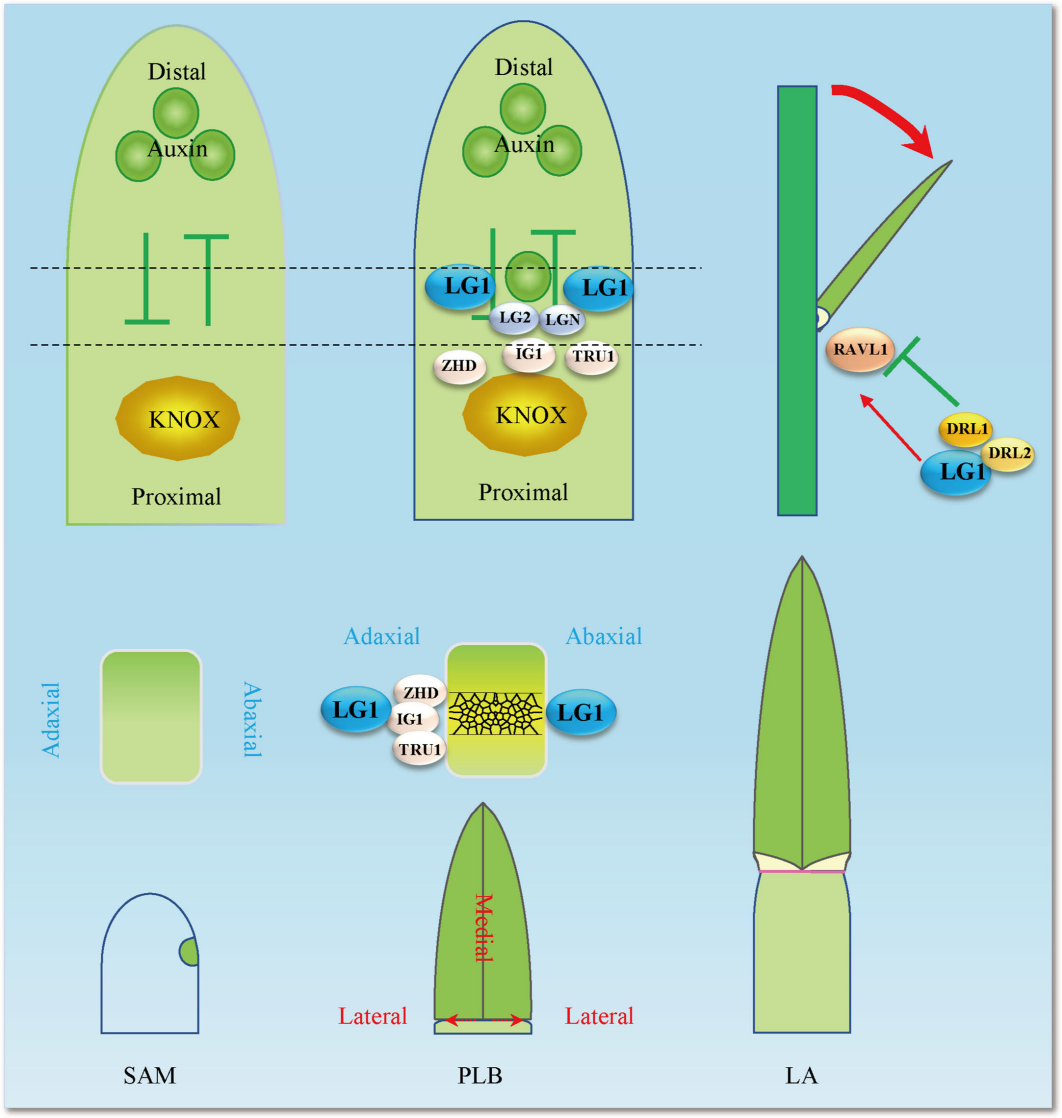
Establishment of LA mediated by auxin and LA-related genes. Auxin and KNOX establish polarity at the distal and proximal ends, respectively, and boundary genes including *ZHD*, *IG1*, *TRU1*, *LG2*, *LGN*, *LG1*, *DRL1*, *DRL2* and *RAVL1* participate in the formation of PLB (preligule band) and the establishment of LA (leaf angle). Black dotted lines indicate the position at the junction of blade and sheath. Red arrows indicate positive regulation, while green lines ended with perpendicular bars represent suppressive regulation.

The *ZmLG1* gene sequence was obtained in 1997 in maize; this gene was found to be expressed at a very low level in the ligular region of leaf primordia, and it may be expressed earlier than the plastochron6 (P6) phase (Becraft et al., 1990; Sylvester et al., 1990; Becraft and Freeling, 1991; Moreno et al., 1997). *ZmLG1* mRNA are expressed autonomously on the adaxial and abaxial PLB at the P6 stage and play a role in signal reception (**Figure 2**). *LIGULELESS2* (*LG2*) is another important gene of the bZIP family and affects the development of the ligular region. Its expression period occurs earlier than does that of *ZmLG1*, and it transmits signals in a noncell-independent manner. Although many studies have shown that *LG1* and *LG2* play a role in the same signaling pathway, there is no actual direct evidence to suggest this relationship (Harper and Freeling, 1996; Walsh et al., 1998; Johnston et al., 2014; Strable and Nelissen, 2021). Later, *ZmLG1* specifically expressed PLB in the P6-P10 stage, and the expression level was found to be higher in P9-P10 than in P6-P7 but not in P1-P5 or in the blades, and *OsLG1* promoter-driven ß-glucuronidase (GUS) expression also showed similar results in rice (Foster et al., 2004; Wang et al., 2021). ZmLG1 affects the positioning and function of *Wavy auricle in blade* (*ZmWab1*) through an unknown pathway, and sheath-like cells appear in the blades of the dominant mutant *Zmwab1*, which is more obvious in *lg1*/*wab1* double mutants (Foster et al., 2004).

**Figure 3:**
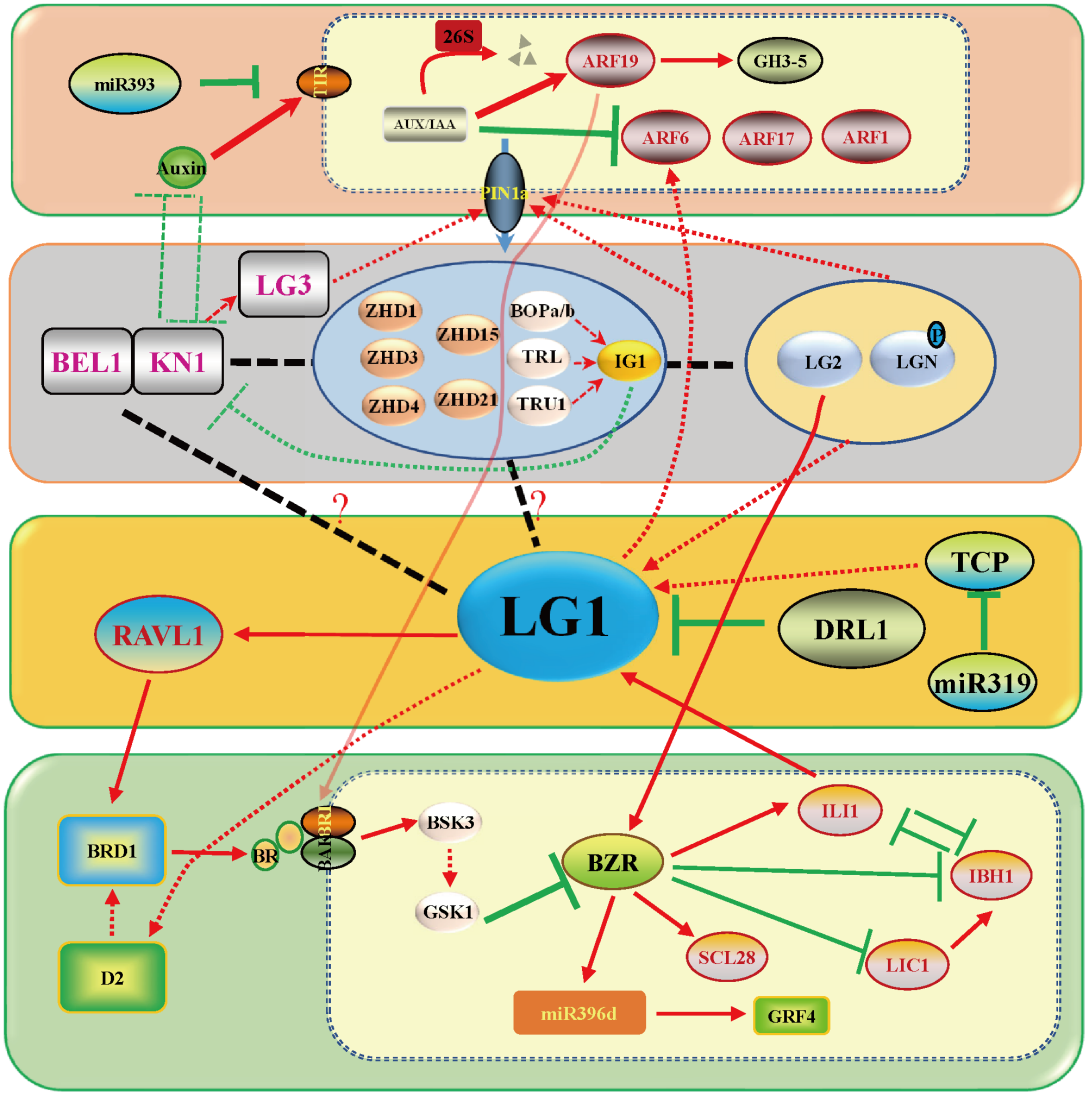
*LG1* participates in the complex LA control network. LG1 regulates *RAVL1* and then participates in the pathway of BR regulating LA by *BRD1*, which includes positive regulation of the LA gene *RAVL1*, *BRD1*, *BRI1*, *BSK3*, *BZR1*, *SCL28*, *ILI1* and miR396, and negative regulation of the LA gene *DRL1*, *IBH1*, *LIC1* and *GRF4*. LG1 is involved in the regulation of LA by auxin and boundary genes including *PIN1a* and *LG2*, although the involvement is not entirely clear. Red arrows indicate positive regulation, while green lines ended with perpendicular bars represent suppressive regulation. The dotted arrows and black lines indicate undetermined relationships.

ZmLG1 was detected using a specific antibody and found to be expressed not only in PLB, but also in the axils of developing tassel branches (Lewis et al., 2014). The *ZmWab1* gene is specifically expressed in flowers, which is necessary for *ZmLG1* function in flower development (**Figure 2**). The specific tassel branch angle decreased in the *Zmwab1* recessive mutant, while the LA remained unchanged. The dominant *ZmWAB1-R/+* mutant has a greater tassel branch angle due to the increased *ZmWAB1* expression level (Lewis et al., 2014). At the same time, *ZmWAB1-R/+* caused abnormal accumulation of ZmLG1, resulting in increased LA. *ZmWAB1* is a member of the TCP family, and the TCP-miR319 module promotes the transition from cell division to cell differentiation in *Arabidopsis*. When TCP expression is high, the TCP-miR319 module inhibits the expansion of the margins, while the miR396-GRF/GIF module of the leaf promotes the expansion of proximal cells (Du et al., 2018; Qin et al., 2022a). The abnormal expression of *ZmWAB1* disrupts the developmental gradient of leaves and changes the expression of *ZmLG1*, thus affecting the growth of leaves (Lewis et al., 2014; Du et al., 2018). Boundary genes such as *ZmLG1* often regulate the development of flowers while regulating the LA. *ZmLG2* has been reported to be involved in tassel branching and the transition from vegetative growth to reproductive growth, and *Unbranched3* (*UB3*), *Tasselsheath4* (*TSH4*) and *LG2* jointly contribute to flower development in a dose-dependent manner (Walsh and Freeling, 1999; Xiao et al., 2022) (**Figure 3**). *OsLG1* is highly expressed in the panicles, sheaths, ligules, leaves and stems of rice, and is related to the control of cell flower development (Zhu et al., 2013) (**Figure 1**). Like that in maize and rice, the expression level of *SevLG1* is also higher in the inflorescences, and this gene is involved in the formation of the lateral organ position boundary and branch meristems in *Setaria viridis* (Zhu et al., 2018) (**Figure 1**). Taken together, these results indicate that *LG1* plays a role in the early development of the ligular region and inflorescence in different species.

## 3 Molecular mechanism of LA regulated by LG1

The LA is the angle formed by the leaves away from the stem to capture sunlight and which is regulated by complex hormones and the external environment (Mantilla-Perez and Salas Fernandez, 2017; Xu et al., 2021). The precise regulation of plant architecture is facilitated by the study of the molecular regulation of LA genes, such as *LG1*, which is extremely important for crop production (Duvick, 2005; Mantilla-Perez and Salas Fernandez, 2017; Wang et al., 2020) (**Table 1**). Phenotypic observations and genetic evidence indicate a role of *LG1* in LA development. In particular, *LG1* is an excellent gene with natural variation affecting LA at the population level, and the molecular regulatory effects of *LG1* on LA, especially the related hormone pathways, have also been discovered in recent years.

**Table 1:** Molecular function of *LG1* and *LG1*-related genes in different species.

Gene name	Species	Gene family description	Molecular Function	Biological Function	Ref.
*ZmLG1* (*Zm00001d002005*)	Maize	SBP	Promotes *RAVL1* expression	Positively regulating LA, inflorescence architecture, ligule and auricle development	(Becraft et al., 1990; Sylvester et al., 1990; Becraft and Freeling, 1991; Harper and Freeling, 1996; Moreno et al., 1997; Lewis et al., 2014)
*AtSPL8* (*AT1G02065*)	*Arabidopsis*	SBP	Promotes GA signal, cell division and differentiation	Positively regulating reproductive development, trichome formation on sepals, and steam filament elongation	(Unte et al., 2003; Zhang et al., 2007; Wang and Wang, 2015)
*OsLG1* (*LOC_Os04g56170*)	Rice	SBP	Inhibit auxin signal, promote cell division and differentiation	Positively regulating LA, inflorescence architecture, ligule and auricle development	(Ishii et al., 2013; Zhu et al., 2013; Wang et al., 2021)
*TaSPL8* (*TraesCS2D01G502900*)	Wheat	SBP	Promotes *TaARF6* and *TaD2* expression	Positively regulating LA, inflorescence architecture, ligule and auricle development	(Liu et al., 2019; Yu, 2019)
*SbLG1* (*SORBI_3006G247700*)	Sorghum	SBP	Unknown	Prediction function is similar to ZmLG1	(Liu et al., 2019; Brant et al., 2021)
*ZmDRL1* (*Zm00001d028216*)	Maize	YABBY	Interfere with the binding of ZmLG1 to Zm*RAVL1*	Negatively regulate LA and midvein development	(Strable et al., 2017)
*ZmDRL2* (*Zm00001d048083*)	Maize	YABBY	Interacted synergistically with ZmDRL1 or ZMM8	Negatively regulate LA and midvein development	(Strable et al., 2017; Du et al., 2021)
*OsDL* (*LOC_Os03g11600*)	Rice	YABBY	Unknown	Negatively regulate LA and midvein development	(Kang et al., 2022)
*SiDPY1* (*Seita.5G121100*)	Foxtail millet	LRR	Attenuates SiBRI1 activity by interacting with SiBAK1	Negatively regulate LA by represses BR signaling	(Zhao et al., 2020)
*ZmRAVL1* (*Zm00001d002562*)	Maize	ABI3VP1/B3	Activates *ZmBRD1* expression	Positively regulate LA, inflorescence architecture, auricle development	(Tian et al., 2019)
*ZmBRD1* (*Zm00001d033180*)	Maize	P450	Promotes gene expression related to BR biosynthesis	Positively regulate LA	(Tian et al., 2019)
*OsCYP85A1* (*LOC_Os03g40540*)	Rice	P450	Promotes gene expression related to BR biosynthesis	Positively regulate LA	(Hong et al., 2002)
*ZmKN1* (*Zm00001d033859*)	Maize	KNOX	Regulate the expression of boundary genes including *ZmLG2* and *ZmLGN*	Blade–sheath differentiation at the midrib region	(Bolduc et al., 2012a; Bolduc et al., 2012b; Johnston et al., 2014; Satterlee et al., 2020; Leiboff et al., 2021)
*OsOSH1* (*LOC_Os03g51690*)	Rice	KNOX	Unknown	Self-maintenance of the SAM, establishment of polarity	(Tsuda et al., 2011)
*ZmLG2* (*Zm00001d042777*)	Maize	bZIP	Positively regulate *ZmBZR1*, *ZmBEH1*and Z*mLG1*	Positively regulating LA, inflorescence architecture, ligule and auricle development	(Harper and Freeling, 1996; Walsh et al., 1998; Walsh and Freeling, 1999; Wang et al., 2022; Xiao et al., 2022)
*OsLG2* (*LOC_Os01g64020*)	Rice	bZIP	Positively regulate *OsLG1*	Positively regulating LA, ligule and auricle development	(Wang et al., 2021)
*OsLG2L* (*LOC_Os05g37170*)	Rice	bZIP	Positively regulate *OsLG1*	Positively regulating LA, ligule and auricle development	(Wang et al., 2021)
*ZmPIN1a* (*Zm00001d044812*)	Maize	PIN	Unknown	Positively regulating formation of PLB	(Carraro et al., 2006; Moon et al., 2013; Johnston et al., 2014)
*ZmWAB1* (*Zm00001d005737*)	Maize	TCP	Positively regulate *ZmLG1*	Positive regulation of LA and tassel branching angle	(Foster et al., 2004; Hay and Hake, 2004; Lewis et al., 2014)
*ZmILI1* (*Zm00001d002121*)	Maize	bHLH	Positively regulate *ZmLG1*	Positive regulate of LA	(Ren et al., 2020)
*ZmUB2* (*Zm00001d031451*)	Maize	SBP	Positively regulate the expression of *ZmTSH1* by interacting with ZmUB3 and ZmTSH4	Positive regulate of tassel branching	(Liu et al., 2021; Kong et al., 2022; Xiao et al., 2022)
*ZmUB3* (*Zm00001d052890*)	Maize	SBP	Positively regulate the expression of *ZmTSH1* by interacting with ZmUB2 and ZmTSH4	Positive regulate of tassel branching	(Liu et al., 2021; Kong et al., 2022; Xiao et al., 2022)
*ZmTSH4* (*Zm00001d020941*)	Maize	SBP	Positively regulate the expression of *ZmTSH1* by interacting with ZmUB2 and ZmTSH3	Positive regulate of tassel branching	(Liu et al., 2021; Kong et al., 2022; Xiao et al., 2022)
*ZmTSH1* (*Zm00001d039113*)	Maize	GATA	Positively regulating the expression of ZmLG2	Positive regulate of tassel branching	(Liu et al., 2021; Kong et al., 2022; Xiao et al., 2022)
*ZmD53* (*Zm00001d023208*)	Maize	SMAX1-LIKE/D53	Positively regulate the expression of *ZmTSH1* by interacting with ZmUB3 and ZmTSH4	Negatively regulate of tassel branching	(Liu et al., 2021; Kong et al., 2022; Xiao et al., 2022)
*ZmZHD1* (*Zm00001d049000*)	Maize	ZF-HD	Unknown	Blade–sheath differentiation	(Leiboff et al., 2021)
*ZmZHD15* (*Zm00001d003645*)	Maize	ZF-HD	Unknown	Blade–sheath differentiation	(Leiboff et al., 2021)
*ZmZHD21* (*Zm00001d041780*)	Maize	ZF-HD	Unknown	Blade–sheath differentiation	(Leiboff et al., 2021)
*ZmTRU1* (*Zm00001d042111*)	Maize	NPR	Activates the expression of *ZmIG1*	Blade–sheath differentiation	(Leiboff et al., 2021; Richardson et al., 2021)
*ZmTRL1* (*Zm00001d011878*)	Maize	NPR	Activates the expression of *ZmIG1*	Blade–sheath differentiation	(Leiboff et al., 2021)
*ZmBOPa* (*Zm00001d004966*)	Maize	NPR	Activates the expression of *ZmIG1*	Blade–sheath differentiation	(Leiboff et al., 2021; Richardson et al., 2021)
*ZmBOPb* (*Zm00001d023246*)	Maize	NPR	Activates the expression of *ZmIG1*	Blade–sheath differentiation	(Leiboff et al., 2021)
*ZmIG1* (*Zm00001d042560*)	Maize	LBD	Repress the expression of *ZmKN1*	Blade–sheath differentiation	(Xu et al., 2016a; Leiboff et al., 2021; Yan et al., 2021)
*ZmGLU1* (*Zm00001d023994*)	Maize	Glycoside hydrolase	Unknown	PLB formation	(Leiboff et al., 2021)
*ZmDHN13* (*Zm00001d033483*)	Maize	Dehydrin protein	Unknown	PLB formation	(Leiboff et al., 2021)

### 3.1 Role of LG1 in LA at the population level

At the population level, the change in LA is determined by the accumulation of small variations in many genes. Many studies have cloned LA genes *via* QTL and GWAS (Tang et al., 2021). According to GWAS analysis, liguleless genes, especially Zm*LG1*, have a greater contribution in upright leaves (Tian et al., 2011). Twenty-five QTLs were identified in a population constructed with Yu82 and Shen137, and QTL *qLA2* was found to harbor a key gene, *ZmTAC1* (*Tilleranglecontrol1*), that regulates LA (Ku et al., 2010; Ku et al., 2011; Ku et al., 2012). Since that discovery, researchers have continuously conducted GWAS or QTL analyses and have identified a large number of LA genes in different populations (Lu et al., 2018; Peng et al., 2021; Tang et al., 2021; Zhang and Huang, 2021). There are 308 QTLs distributed across maize chromosomes, of which chromosome 1 contains the most 63 (QTLs), while chromosome 6 contains a minimum of 15 QTLs. These QTLs are divided into 17 important intervals, including 7 reported LA regulatory genes, such as *ZmDRL1*, *ZmDWF* and *ZmLG1*. A large number of QTL hot spots exist in which the target genes have not been identified (Qin et al., 2022b). Two important QTL sites, *UPA1* and *UPA2*, have been identified from a population obtained by crossing maize and its ancestor teosinte. *UPA2* is located 9.5 kb upstream of *ZmRAVL1*, which is a binding site of ZmDRL1/2. ZmLG1 activates the expression of *ZmRAVL1*, and the interaction between ZmDRL1/2 and ZmLG1 inhibits the activation of *ZmRAVL1*(**Figures 2**, **3**). This study reveals the regulatory network of maize LA from the perspective of domestication and opens the door to the transformation from molecular breeding to application (Tian et al., 2019). QTL/GWAS analysis has suggested that *ZmLG1* plays an important role in regulating LA in the population. How to identify more loci and complex remote regulatory mechanisms requires further research, especially involving QTLs with specific leaf positions, which deserve more attention (Tang et al., 2021).

### 3.2 Molecular regulatory effects of LG1 on hormone-regulated LA

Much research has been done on the regulation of LA by plant hormones. Auxin represents the basis for promoting the formation of PLB and its content is thought to have a negative role in LA. BRs have a more significant function in LA, and a large number of genes involved in BR synthesis and signaling pathways participate in regulating LA. In recent years, it has been found that *LG1* participates in the auxin and BR pathways to regulate LA. Two well-defined regulatory pathways are known, namely, DRL1/2-LG1-RAVL1 and ILI1-LG1, which are involved in BR signaling, and two poorly understood pathways, KN1-LG2-LG1-ARF and TCP-LG1-ARF, are known to be involved in auxin signaling (Xu et al., 2021; Cao et al., 2022b) (**Figure 3**).

Hormones play an important role in the whole process of plant growth and development. At the early stage, auxin accumulates at this position of leaf primordium initiation in the shoot apical meristem (SAM) (Bolduc et al., 2012a). The growth polarity of proximal–distal patterning is established between KNOX and auxin. The boundary gene represented by *ZmLG1* plays a role in the connection. *Zmlg1* mutants are accompanied by changes in the expression of *ZmPIN1a* (Bolduc et al., 2012a; Johnston et al., 2014). Auxin is an important signal for the development of veins and promotes the development of tertiary veins in leaves (Robil and McSteen, 2022). It is still unclear whether auxin signaling to *ZmLG1* participates in the PLB formation process from the midvein to the leaf margin. At present, auxin is located distal to the leaf primordium, *ZmKN1* is located proximal to the leaf primordium, and boundary genes such as *ZmLG1*, *ZmLG2*, *ZmBell* and *ZmBOP* are located at the junction promote the formation of the ligular region (Carraro et al., 2006; Osmont et al., 2006; Johnston et al., 2014; Richardson and Hake, 2018; Toriba et al., 2019). Combined with the transcriptome data including that of *Zmlg1* at the PLB early stage, a gene coexpression network was constructed. The network module of *ZmLG1* includes 270 genes (Johnston et al., 2014; Leiboff et al., 2021). According to this coexpression network, a model of the early transcriptomic proximodistal prepattern was proposed. At the bottom of leaf primordia, ZmKN1 regulates downstream genes including *ZmZHDs*, *ZmBOPs*, ZmBOPs, activating the expression of the *ASYMMETRIC LEAVES2* (*AS2*)-related gene (*INDETERMINANT GAMETOPHYTE1*, *IG1*). *ZmLG1*, *ZmLG2*, *ZmLGN* and *ZmRAVL* are coexpressed in PLB, and previous genetic evidence also shows that they are in the same signaling pathway. The establishment of this development gradient provided a basis for the subsequent formation of LA (Leiboff et al., 2021). According to the present data, there is a ZmKN1-ZmLG2-ZmLG1 pathway closely related to auxin, which is affected more by the gene regulation that regulates LA (**Figure 3**). The interaction of OsGRF7 and OsGIFs promotes an increase in auxin content and inhibits the LA size in rice (Chen et al., 2020), and miR393 inhibits the expression of *OsTIR1* and *OsAFB2* to promote LA size (Bian et al., 2012).

*TaSPL8*, a homologous gene of *ZmLG1* (**Figure 1**), directly activates the expression of *TaARF6*, which is a core auxin signaling pathway gene that regulates LA. The TaSPL8*-*TaARF6 pathway provides the possibility for *ZmLG1* to regulate LA through the auxin signaling pathway in a similar way (Liu et al., 2019) (**Figure 3**). The maize seedling indoleacetic acid (IAA) concentration gradient showed a significant difference between the wild type and *Zmlpa1* mutant after treatment with exogenous 100 µmol•L^–1^ IAA (Ji et al., 2022). Nonetheless, the response of the *Zmlg1* mutant to exogenous auxin treatment has not been reported and needs to be explored. As a member of the SBP family, the *LG1* homolog has also been reported to be related to auxin in rice. OsSPL14 of the SBP family binds to the promoter of *OsPIN1b* to regulate the distribution of auxin and inhibit the growth of tillers (Li et al., 2022). Functioning upstream of *ZmLG1*, two *ZmLG2* homologous genes, *OsLG2* and *OsLG2L*, were identified to redundantly regulate the LA of rice. Moreover, the expression level of auxin signal-related genes was increased in *OsLG1-cri*, *OsLG2-cri* and *OsLG2L-cri* knockout mutant (Wang et al., 2021). Therefore, it was proposed that there was a quantitative effect at play to regulate the LA in the same signal pathway between *OsLG1* and *OsLG2*/*2L* in rice. *OsLG1* and *OsLG2*/*2L* negatively regulate the expression of auxin-related genes to promote ligular region division and development (Wang et al., 2021). The auxin-related genes *GH3.6*/*DFL1*, *axi1-like* and *PIN5* and the TF-encoding genes *ARF6*, *ARF9* and *ARF16* were highly expressed in the PLB, and their expression levels decreased in the *Zmlg1* mutant. These are potential ZmLG1 downstream regulatory genes (Johnston et al., 2014). The auxin signal-related genes *OsARF19*, *OsARF6*, *OsARF11* and *OsARF17* were also reported to participate in the development of the LA of rice (Zhang et al., 2015; Liu et al., 2018; Huang et al., 2021). On the other hand, ZmKN1 directly binds and regulates the expression of *ZmLG2*, and the KN1-LG2-LG1-ARF pathway regulates LA (Bolduc et al., 2012b; Liu et al., 2019; Wang et al., 2021). The regulation of *ZmWAB1*, a TCP family member, is redundant in LA. *Zmwab1* does not exhibit a reduced-LA phenotype, but overexpression of *ZmWAB1* can ultimately increase LA by increasing the expression of *ZmLG1* (Lewis et al., 2014). Therefore, there is a way for the TCP-LG1-ARF pathway to alter the LA, but other TCP members involved need to continue to be identified.

BRs constitute one of the most critical hormones affecting plant architecture. A large number of genes related to LA are directly or indirectly involved in BR biosynthesis and signaling pathways (Mantilla-Perez and Salas Fernandez, 2017; Gruszka, 2020; Li et al., 2020; Nolan et al., 2020; Xu et al., 2021; Cao et al., 2022b). There are no reports about exogenous BR treatment in the *Zmlg1* mutant, but it can be determined that *ZmLG1* is closely related to BR signaling. Through experimental identification, it was found that ZmLG1 directly binds to the promoter of *ZmRAV1*, and ZmRAVL1 binds and activates the expression of the downstream gene Zm*BRD1*, which is the key BR biosynthesis-related gene (Tian et al., 2019). Although *ZmRAV1* functions downstream of ZmLG1, the *Zmravl1* mutant does not show defective ligules, but the width of the ligular band and the mature auricle margin decrease, and the number of adaxial sclerenchyma cell layers increases (Tian et al., 2019). *ZmRAV1* is one of the many redundant regulatory genes that functions downstream of ZmLG1, which indicates that the regulation of LA not only is controlled by the genes in the early stage but also is important in the later stage. This small change can realize the application of genes in breeding without affecting other traits. In addition, ZmLG1 interacts with ZmDRL1 to inhibit the function of ZmLG1 activation of *ZmRAVL1*(Tian et al., 2019). *ZmDRL1* belongs to the YABBY family and promotes the development of the midvein of the leaf blade. *Zmdrl1* mutations lead to an increase in LA as leaves were noticeably droopy because the strength of leaves midribs was reduced(Strable et al., 2017). SiDPY1, an LRR receptor-like kinase in *foxtail millet*, interacts with the BR signaling receptor SiBAK1 and negatively regulates SiBRI1 (Zhao et al., 2020). Therefore, DRL1-LG1-RAVL is involved in the pathway by which BRs regulate LA. Functioning as a downstream gene of OsBZR1 in BR signaling, *Increased Lamina Inclination1* (*OsILI1*) positively regulates the LA of rice. At the same time, OsILI1 interacts with ILI1 binding bHLH1 (OsIBH1) to jointly regulate LA (Zhang et al., 2009). In maize, ZmILI1 binds specifically to the promoter (-1868 bp) of *ZmLG1* and activates its expression (Ren et al., 2020). *ZmIBH1-1* has a negative regulatory effect on LA by mapping. In addition, through CRISPR−Cas9-mediated genome editing in *Setaria*, the effects of its homologous gene *Sv.IBH1-1* and *Sv.IBH1-2* result in a phenotype of increased LA (Cao et al., 2020). Therefore, we propose a hypothesis that there may be an interaction between ZmILI1 and ZmIBH1 to jointly regulate the expression of *ZmLG1*. There is additional evidence that *ZmLG1* participates in the BR pathway. In wheat, TaSPL8 directly binds to GTAC the core sequence in the *TaD2* promoter, and activates *TaD2* expression (Liu et al., 2019). Therefore, *ZmLG1* possibly participates in the BR pathway in a conservative way like *wheat* TaSPL-TaD2. A recent study showed that another important LA gene, *ZmLG2* also participates in the BR signaling pathway. ZmLG2 directly combined with and activated the expression of the downstream genes *ZmBZR1* and *ZmBEH1*, further activating the expression of the downstream *ZmSCL28* to regulate LA (Wang et al., 2022). According to previous studies, *ZmLG1* and *ZmLG2* participate in the same pathway (Harper and Freeling, 1996; Moreno et al., 1997; Tian et al., 2019), so *ZmLG1* participating in the complex process of BR signaling is reasonable (**Figure 3**).

## 4 Molecular regulation of *LG1* in the flower development

**Figure 4:**
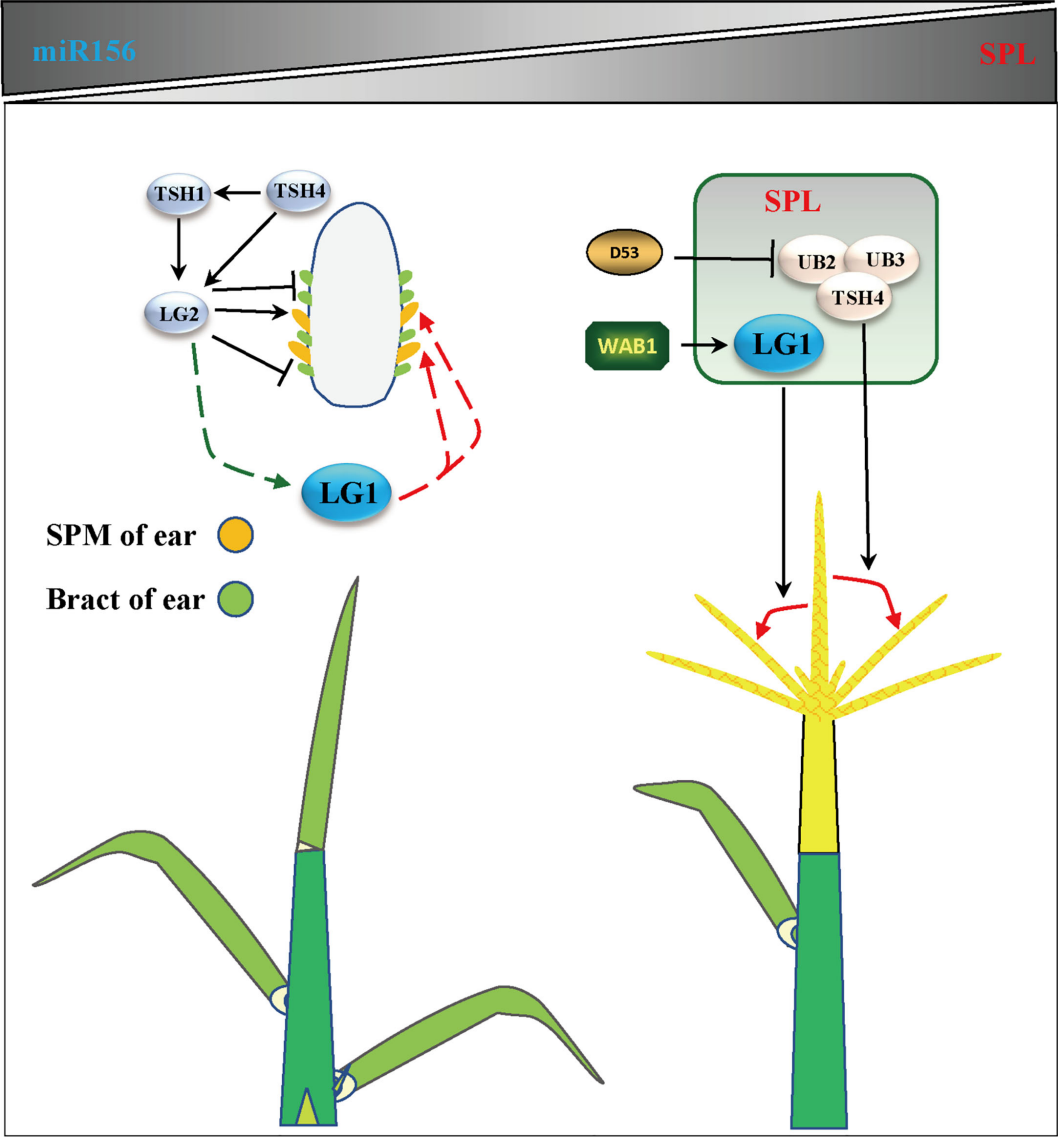
*LG1* participates in flower architecture. Genes related to tassel branching or ear development include *LG2*, *TSH1*, *TSH4*, *UB3*, *UB2*, *WAB1* and *D53*, which participates in the regulation of flower architecture of LG1 with aging. *LG1* may be regulated by LG2 and participate in the development of SPM (spikelet pair meristem) or bract in ear. *LG1* regulated by WAB1 and participate in the development of tassel branching. The triangle vertical gradual shadings underneath miR156, SPL genes indicate expression level changes with age, LG1 cooperates with miR156-targeted SPLs to regulate tassel branching. Black arrows indicate positive regulation, while black lines ended with perpendicular bars represent suppressive regulation. Red and green dotted lines arrows indicate undetermined relationships.

**Figure 5:**
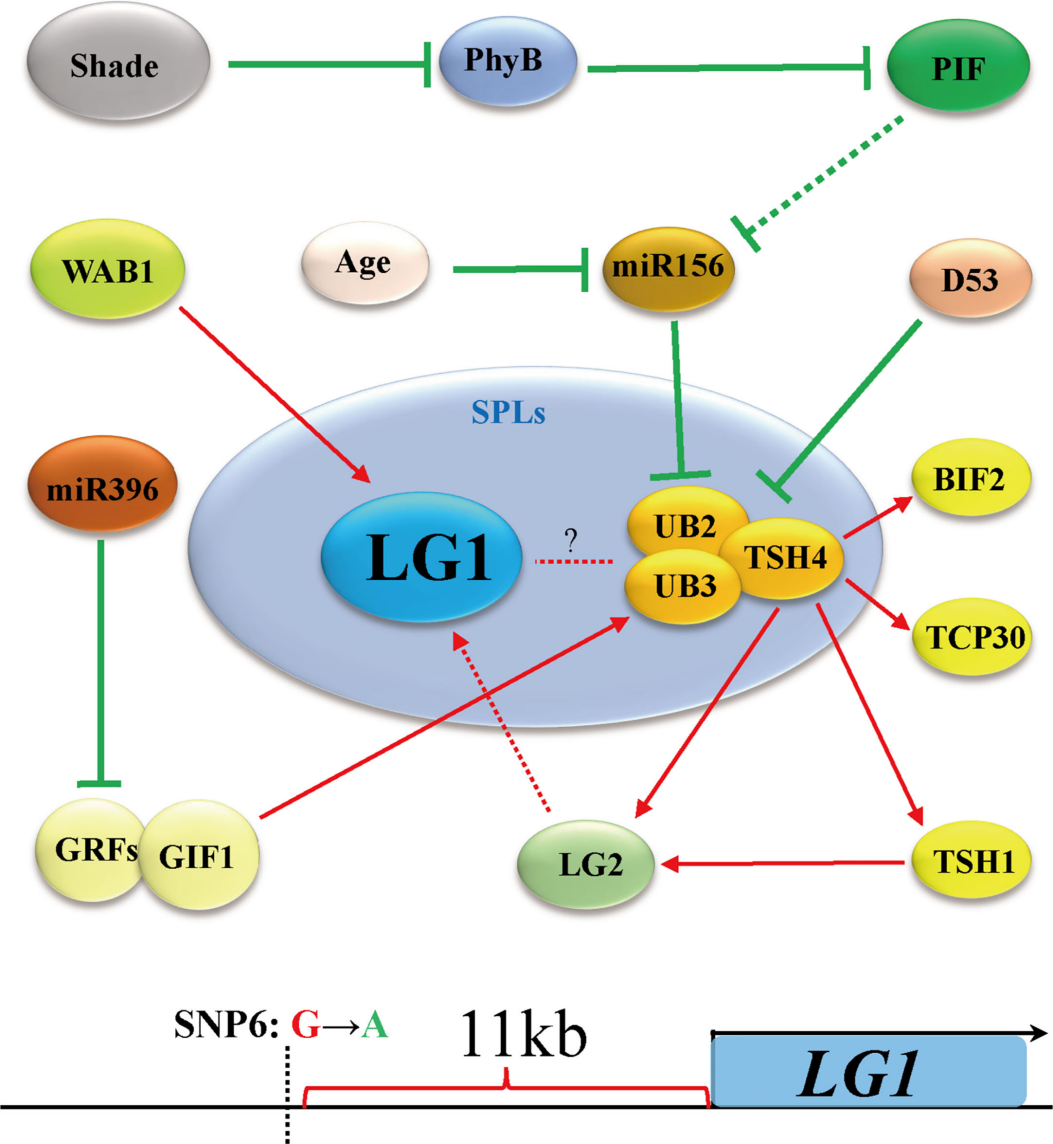
*LG1* may participate in the regulation of flower architecture *via* the shade pathway. Shading negatively regulates miR156 through *PHYB* and *PIF*, thereby promoting the expression of *UB2*/*UB3*/*TSH4*. *LG1* may participate in this way downstream of LG2. The *OsLG1* promoter contains an important element for regulating flowers architecture at a long distance. Red arrows indicate positive regulation, while green lines ended with perpendicular bars represent suppressive regulation. Red and green dotted lines arrows indicate undetermined relationships.

Flowering is the most important feature for plants to adapt to the environment, and is the characteristic of transformation from vegetative growth to reproductive growth (Walsh and Freeling, 1999). Due to the different sunshine durations at different latitudes of the earth, plants require a specific time to flower. Therefore, plants have difficulty adapting to the local environment due to changing latitudes, showing delayed or even an inability to flower (Huang et al., 2018). Increasing evidence shows that members of the SBP/SPL family play an important role in the transformation from vegetative growth to reproductive growth. The expression of *SPLs* is inhibited by microRNA156s (miR156s) at the vegetative growth stage, the expression of miR156s decreases with age, and the increase in *SPL* expression is the main process of reproductive transformation (Unte et al., 2003; Preston and Hileman, 2013; Wang and Wang, 2015) (**Figure 4**). In *Arabidopsis*, the *ZmLG1* homolog *AtSPL8* is necessary for fertility during spore development in a gibberellic acid (GA)-dependent manner (Zhang et al., 2007; Xing et al., 2010; Wang and Wang, 2015). In rice, *OsLG1*, which affects the angle of panicle branches, has also been reported. An *SPR3* locus including a 9.3 kb genomic region of the *OsLG1* promoter regulates the branch angle of the panicle and seed shattering (Ishii et al., 2013). Subsequently, through the use of multiple inbred lines, it was found that the region controlling panicle architecture was a 3.3 kb region 10 kb upstream of the *OsLG1* translation start site, and the variation of one single-nucleotide polymorphisms6 (SNP6) might affect panicle architecture by changing the methylation level (Zhu et al., 2013) (**Figure 5**). In maize, *ZmLG1* affects panicle architecture, but it is still unclear whether there is a regulatory mechanism at play similar to that of *OsLG1* upstream of the promoter (Lewis et al., 2014). However, it has been confirmed that *ZmLG1* genetically regulates panicle architecture through *ZmWab1* (a member of the TCP family). Considering the conservation of the SPL family in plants, we speculate that ZmWAB1 may bind to a region upstream of the *ZmLG1* promoter to regulate the panicle architecture of maize (Wang and Wang, 2015; Xu et al., 2016b). *ZmLG2*, another key gene that regulates LA, plays an important role in the transformation from vegetative growth to reproductive growth, and the number of panicle branches is reduced in the *Zmlg2* mutant (Walsh and Freeling, 1999). Together, with *AtBOP* and *AtROXY*, the *ZmLG2* homologous gene *AtTGA9* cooperatively regulates pollen fertility (Hepworth et al., 2005; Li et al., 2009; Murmu et al., 2010). In maize, the SPL family genes *UB2*, *UB3*, *TSH4* and *LG2* form a pathway to inhibit the growth of bracts and increase the number of inflorescence branches (Liu et al., 2021; Xiao et al., 2022) (**Figure 4**). Through simulated shade experiments, it was found that *UB2*, *UB3* and *TSH4* constituted the core of the shade response and regulated the expression of the downstream genes *Barren inflorescence2* (*BIF2*) and *Zea mays teosinte branched1/cycloidea/proliferating cell factor30* (*ZmTCP30*) (Kong et al., 2022). In addition, *UB3* is also regulated by GIF1 and affects ear development (Zhang et al., 2018). Whether LG1 is involved in this process is still unclear, but the earliest expression of *SevLG2* can be identified in inflorescence development in *Setaria viridis*, and *SevLG1* expression regulates the development of flowers (Zhu et al., 2018).

## 5 Perspectives

Plant architecture is very important for crop production. Erect leaves can make full use of light energy to achieve yield increases by dense planting, increasing light penetration to the lower canopy, improving airflow as well as heat distribution (Duvick, 2005; Mansfield and Mumm, 2014; Ort et al., 2015; Wang et al., 2020). *LG1*, a major gene that regulates LA, has a conservative function in plants and is highly valuable for molecular breeding (Becraft et al., 1990; Sylvester et al., 1990; Moreno et al., 1997; Foster et al., 2004; Osmont et al., 2006; Lee et al., 2007; Ahn et al., 2008; Zeng et al., 2009; Liu et al., 2019; Yu, 2019). Much evidence shows that *LG1* is involved in the pathway of regulating LA by hormones such as auxin and BRs and is the core regulator of LA and inflorescence development (Moon et al., 2013; Johnston et al., 2014; Okagaki et al., 2018; Liu et al., 2019; Tian et al., 2019; Ren et al., 2020; Dou et al., 2021; Guo et al., 2021). Other hormones, including GA, cytokinin (CK), ethylene (ET), directly or indirectly affect BR synergetic regulation of LA (Li et al., 2020; Cao et al., 2022b). Therefore, there is still much room to explore the *LG1* regulatory network related to the development of other ligular regions in plants (Cao et al., 2022a; Liu et al., 2022). As a member of the SBP family, *LG1* shows essential functions in reproductive transformation, especially in flower development (Wang and Wang, 2015; Xu et al., 2016b). Nonetheless, further exploration of the function of *ZmLG1* and genetic experiments are needed.

Based on the development of the *LG1* and SBP families in LA and flowers, we propose several problems need of solving. First is the source and signal of the development of PLB. Although transcriptome data are available for the early stage of PLB development and concerning the antagonistic effects of auxin on the distal and proximal KNOX families in leaf primordia, the transmission signal of PLB and the *LG1*-specific receiving signal pathway is still unclear (Johnston et al., 2014; Leiboff et al., 2021). Recently, according to information obtained from developmental genetics and computational models, there has been a new discussion on the origin of sheaths in plant species, and the petiole-sheath hypothesis has been proposed (Richardson et al., 2021). Similarly, exploring the precise formation process of PLB by the use of the model could help us understand the development mechanism of PLB more in depth. Another feasible method is to accurately analyze the changes in PLB development through single-cell technology, especially single-cell analysis in maize SAM development, which provides one possible direction (Satterlee et al., 2020). Second is, the *LG1* regulatory network and its response to the external environment. *LG1* is involved in BR signaling through *RAVL1* in regulating LA, but there is still no research on whether *LG1* responds to BRs and whether it participates in the regulation of the BR biosynthesis pathway (Liu et al., 2019; Tian et al., 2019) With respect to the SPL family, it is proposed that light signaling may be associated with miR156-SPL through the PHYB-PIF pathway, but whether *LG1* participates in this process needs further experimental verification. It is also unclear whether DRL1/2 interacting with LG1 participates in PLB signal initiation, although BR signaling can be suppressed through SiBAK1 interaction with SiDPY in *Setaria* (Strable et al., 2017; Wei et al., 2018; Tian et al., 2019; Zhao et al., 2020). Third, the application of *LG1* in breeding. Because of its extremely severe phenotype, *ZmLG1* has not been availably applied in molecular design breeding. There are SNP near the 10 kb upstream region of *OsLG1* that regulate flower development in rice, so it is reasonable to believe that they are similar in maize, and even precisely control the LA *via* different loci (Ishii et al., 2013; Zhu et al., 2013). Recently, a practical strategy has emerged. The promoter of *OsIPA1* (*Ideal Plant Architecture 1*), which is a member of the SBP family, was screened in a high-yield variety that could break the linkage between favorable and unfavorable traits to obtain the ideal plant architecture through tiling-deletion-based CRISPR–Cas9 screening technology, which also provides a new idea for *ZmLG1* in the future (Song et al., 2022). Therefore, we should make full use of new technologies and means to explore the functions of *LG1* and its potential value.

## Author contributions

HZ and LQ designed and drafted the work. All authors approved the final version of the manuscript.
